# Self-ordered silver nanoparticles on nanoconcave plasmonic lattices for SERS multi-antibiotic detection

**DOI:** 10.1515/nanoph-2025-0108

**Published:** 2025-06-26

**Authors:** Gohar Ijaz Dar, Elisabet Xifre-Perez, Lluis F. Marsal

**Affiliations:** Departament d’Enginyeria Electrònica, Elèctrica i Automàtica, 16777 Universitat Rovira i Virgili, Avinguda Països Catalans 26, 43007, Tarragona, Spain

**Keywords:** nanoparticles, SERS, nanoconcave plasmonic lattices, sensing, tetracycline, amoxicillin

## Abstract

Antibiotic detection at trace levels in different matrices is an important tool for environmental monitoring, clinical diagnostics, and pharmaceutical quality control. Using aluminum concavities covered with silver nanoparticles (AgNPs), this study introduces another approach for the surface-enhanced Raman spectroscopy (SERS) detection of antibiotics. The optimal substrate provided by the aluminum concavities and the outstanding plasmonic enhancement of the AgNPs greatly enhances the adsorbed Raman signals of the antibiotic molecules. First, we used a controlled magnetron sputtering technique to deposit AgNPs onto the SERS substrates, synthesized by anodizing aluminum into highly organized concave dimensions. Detection limits approaching the 10^−10^ M concentration level, owing to an EF of 10^8^, proved that these substrates successfully detected various antibiotics, including amoxicillin and tetracycline. An in-depth examination of the SERS spectra revealed distinctive peaks that correspond to functional groups, allowing for the exact identification and quantification of the antibiotic compounds. The synergistic impact of the aluminum concavities and silver nanofractals results in extremely homogenous substrates that are reproducible and sensitive.

## 1 Introduction and background

The use of antibiotics has been widespread in many areas to prevent and treat various bacterial diseases. Antibiotics are efficient against multiple Gram-negative and Gram-positive bacteria, and their efficiency ranges from mild to effective [1]. They can be taken orally and are typically administered to patients for bacterial infection treatments. After going through the metabolism process in the body’s tissues, antibiotics are mostly removed as the substance originally in the urine [2], [3]. The most effective treatment typically involves using several different antibiotics in combination. Amoxicillin (Amx), ciprofloxacin (CIP), and tetracycline (Tc) are the antibiotics that fall under this category [4], [5].

The widespread use of antibiotics in medicine, animal feeding, and agriculture has ramped up to dangerous [6], [7]. Inappropriate and excessive use of these drugs can be linked to a wide range of adverse health effects in human beings, such as the suppression of bone marrow, toxicity to the liver, carcinogenicity, and genotoxicity [8], [9], [10], [11]. Also, the development of antibiotic resistance, immunological weakening, and cancer are among the many severe health effects linked to the ingestion of antibiotics [12], [13]. The danger of overusing antibiotics has become such a major problem that even many countries have banned or severely limited some of them. For this reason, it is of the utmost importance to monitor many antibiotics in plasma, serum, whole blood, and urine on time, effectively, and simultaneously to provide appropriate instructions for using antibiotics [14], [15], [16].

A significant obstacle has emerged in this regard. Traditional technologies such as capillary electrophoresis and liquid chromatography coupled with various devices have been widely employed for antibiotic detection up till now due to their high selectivity and sensitivity [17], [18]. Antibiotic detection methods are now used in the lab, mostly gas chromatography (GC), high-performance liquid chromatography (HPLC), and LC with the use of mass spectrometry (LC-MS) [19], [20], [21]. These methods are labor-intensive and expensive; experts must complete them consistently and precisely. Hence, creating novel methods for detecting antibiotics that must be quick, accurate, dependable, and less complex is a significant subject. As a novel trace analysis technique for detecting target molecules from their “fingerprint-like” spectra, surface-enhanced Raman spectroscopy (SERS) has garnered much interest from academics across disciplines in recent years. Surface-enhanced Raman scattering (SERS) is a powerful method for molecular sensing that has found applications in many different areas, including electrochemistry, materials science, biochemistry, biosensing, and catalytic chemistry [22], [23], [24], [25]. Using ultrathin anodic aluminum oxide (AAO) membranes, the centimeter-scale plasmonic nanoarray was created on a quartz substrate. Liquid surface-enhanced Raman spectroscopy (SERS) was used as a real-time and *in situ* tool to investigate the oxidation mechanisms in plasmonic catalysis, using the decarboxylation of p-mercaptobenzoic acid (PMBA) as a case study [26]. A metallic surface’s architecture greatly affects the strength and distribution of the SERS signal; metals like gold, silver, copper, and similar substances are commonly used for Raman enhancement [27], [28]. Recently, a wide range of SERS substrates has been developed to identify species’ chemical structures, including nanoparticles, nano stars, nanopyramids, nanorods, and hybrid materials [29], [30]. It is extremely challenging to work with nanostructures because of toxicological concerns, coagulation, and the difficulty of following up in physical systems. The non-uniform aggregation of nanostructures also makes the SERS signal repeatability poor. A perfect SERS substrate would not only have an excellent SERS profile, but it would also eradicate the issues related to using nanopatterns.

With the inelastic light scattering’s distinctive, narrow, well-resolved bands, SERS can detect multiplexes and provide extensive vibrational information on the adsorbed molecules. This method has been investigated for Tc detection in many samples [31], [32]. One example is the work of Li et al., who used Au hollow spheres to detect Tc with a detection limit as low as 0.1 g/L [33]. The use of a SERS substrate consisting of gold NPs encouraged Y. Xie et al. to detect antibiotic residues, including furadantin, furaltadone, and mixtures of the two, in aquaculture water and aquatic products with a limit of detection (LOD) of 5 mg/L [34]. In another research study, Y. Zhang and colleagues investigated the limit of detection (LOD) for enrofloxacin, furazolidone, and malachite green in fish products using the commercial SERS substrates Klarite or Q-SERS. They achieved a LOD as low as 800 μg/L [35]. J. Song and colleagues documented the LODs ranging from 0.01 μg/L to 0.1 mg/L after examining the detection of malachite green, crystal violet, furazolidone, and chloramphenicol employing a silver nanowires SERS substrate [36]. However, in most cases, the findings from these and similar studies are obtained by studying how a single target molecule affects a Raman band’s peak intensity, band location, and FWHM. Therefore, limitations occur when the sample framework is overly complex, for instance, when the mixture contains multiple target substances which are not easily detectable and identified using traditional peak analysis methods like peak deconvolution and curve fitting [37]. Hence, scientists are creating novels, very effective SERS substrates by utilizing them directly with quick implementations. However, owing to the irregular distribution of nanoparticle (NP) sizes, the management of interparticle spaces is quite complex, along with aggregation. Another study used a wearable surface-enhanced Raman scattering (SERS) device with integrated plasmonic trimers and a hydrogel membrane for non-invasive sweat uric acid monitoring [38]. According to Hao et al., the plasmonic Au@Al_2_O_3_–Au–Au@Al_2_O_3_ trimers, which are made up of two dimer particles and a trap particle in between, can selectively induce target molecules into the traps where “hotspots” are located, achieving a detection limit of 10^−14^ M of 4-methylbenzenethiol, 4-mercaptopyridine, and 4-aminothiophenol [39]. Nonetheless, the electrostatic interactions of the metal NPs may influence the measurement results, leading to an inconsistent SERS signal [40].

According to a recent study, a novel SERS substrate based on V_5_S_4_ nanopompons was developed. It exhibited the use of AI and SERS technology to detect antibiotics, including ciprofloxacin, tetracycline, and chloramphenicol in milk, and it obtained a limit of detection of 10^−11^ M for Rhodamine 6G (R6G) [41]. A recent study found that silver-functionalized silicon nanowire substrates can detect the antibiotic ceftriaxone in spiked fresh plasma and microdialysis samples, with a limit of detection of 1.4 μM in microdialysate samples [42]. Herein, we present the simple approach for creating Ag NPs using nanoporous anodic aluminum oxide (NAA) template-assisted electrochemical deposition to make the SERS substrate simple, inexpensive, reproducible, scalable, and surfactant-free. To create concave Al substrates, we chemically etched the Alumina and removed the porous structure. Consequently, Ag coating is carried out by magnetron sputtering into prepared Al nanoarrays. After Ag sputtering, we thermal annealed the prepared Ag-coated Al concavities, as shown in Schematic 1. Our silver nanoparticle-decorated aluminum concavities offer a special and very efficient SERS detection substrate. High sensitivity and reproducibility are achieved through the synergistic interaction of silver nanoparticles with aluminum nanopits, which strengthens the local electromagnetic field. Our work highlights the adaptability and usefulness of our SERS substrates by successfully detecting amoxicillin and tetracycline in a variety of matrices. Our method’s practical applicability is further demonstrated by its capacity to identify various antibiotics in mixes without experiencing severe interference.

**Schematic 1:**
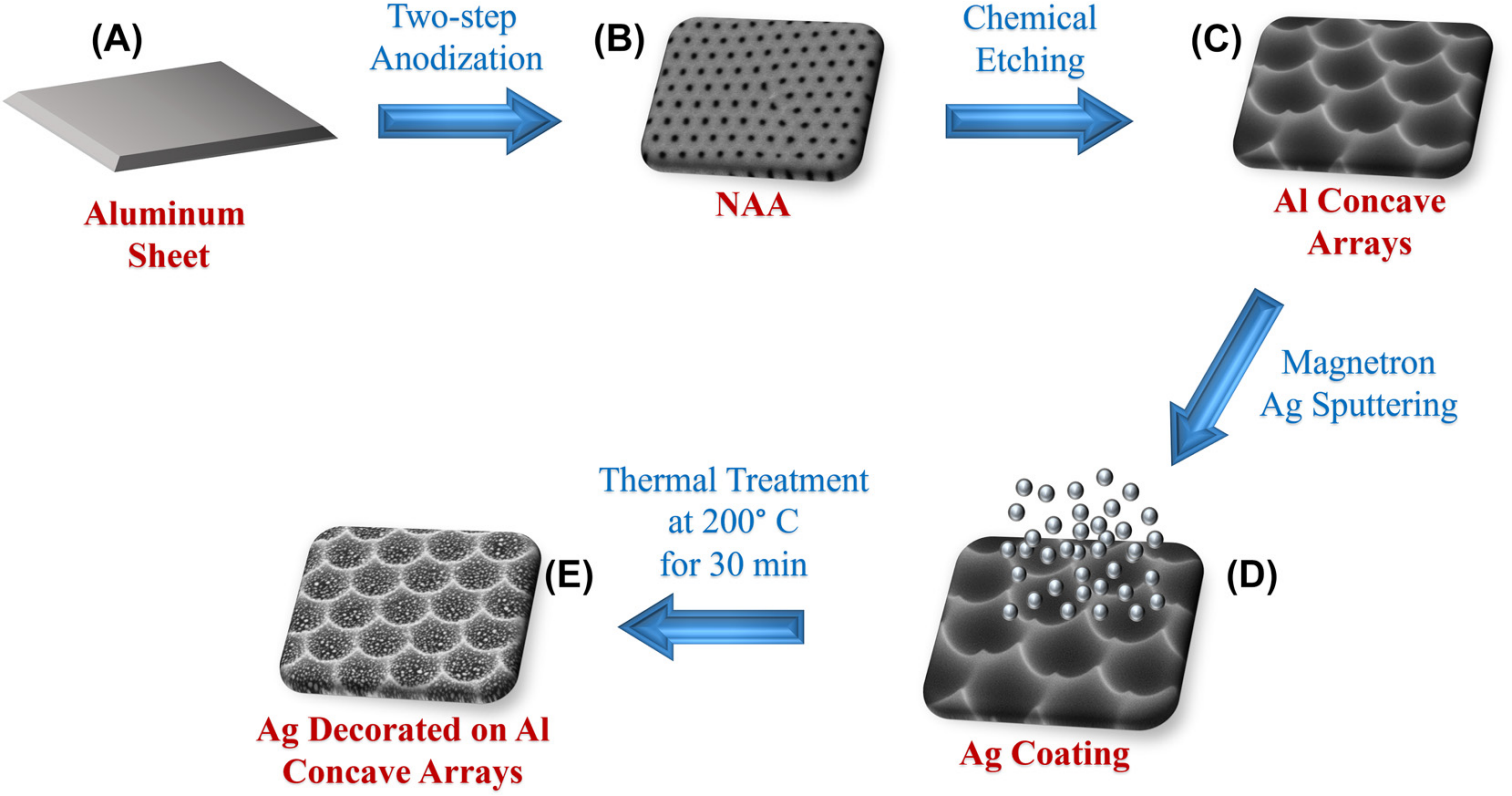
Schematic illustration of the synthesis of Ag-enriched Al nanoconcavities for SERS activity. (A) The aluminum sheet is clean and ready for anodization. (B) performance of a two-step anodization by using phosphoric acid as an electrolyte. (C) Chemical etching is used to get the desired Al nanoconcavities. (D) Ag magnetron sputtering on the Al nanoconcavities for 180 s. (E) thermal annealing treatment at 200 °C for 30 min to achieve the NPs on the Al nanoconcavities.

## 2 Experimental section

### 2.1 Fabrication of Al nanoconcavities substrates

High-purity aluminum foils, with a purity of 99 %, were systematically cut into 2 × 2 cm^2^ samples, ensuring they were not bent at any angle. Then, the Al samples were mechanically polished, ultrasonicated in water and acetone to eliminate contaminants and roughness, and thoroughly dried. The samples were subjected to electrochemical polishing on one side using a mixture of perchloric acid and water (in a ratio of 1:4) at a temperature of 3 °C for 4 min . The electropolishing method involves the application of a voltage of 20 V to a two-electrode setup, with a copper plate serving as the cathode. Once the Al samples were thoroughly rinsed with water and ethanol and dried using compressed air, they were prepared for further anodization procedures.

The nanoporous anodic aluminum oxide (NAA) templates were produced by performing a two-step anodization procedure using a phosphoric acid electrolyte (1 wt%) under ramping voltage conditions [43]. The initial anodization aims to create a self-organized porous layer using high voltage settings (195 V). To overcome the several elements that cause breakdown and achieve a uniform pattern of pores on the aluminum foils, it is necessary to establish an oxide film’s growth before reaching the ramping voltage up to 195 V. The protective oxide layer was created using phosphoric acid under mild anodization conditions, with a voltage of 175 V, at a temperature of −10 °C for 3 h . Subsequently, the voltage is incrementally raised at a consistent rate of 0.005 V per second, starting from 175 V and reaching a maximum of 195 V, remaining for an additional 15 h [44], [45]. Due to the high applied voltage, it is necessary to maintain a very low temperature (−10 °C) to prevent the burning of samples under the critical circumstances of anodization.

**Figure 1:**
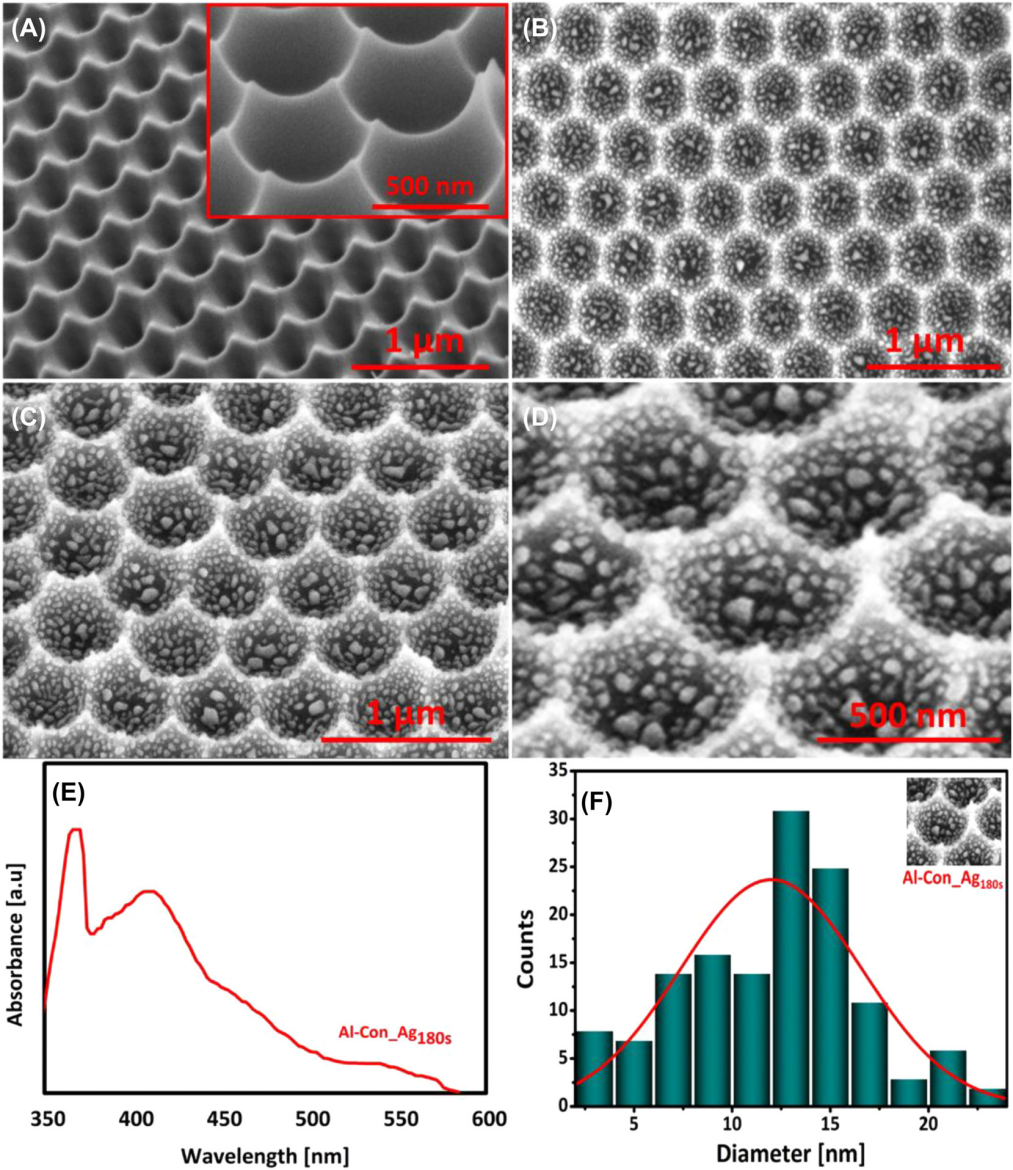
FESEM images (A) NAA are synthesized by a two-step anodization process with phosphoric acid as an electrolyte. Inset: Al nanoconcavities substrate obtained after the chemical removal of the NAA layer. (B) Top view of Al nanoconcavities substrate enriched with Ag NPs after 180 s of sputtering and thermal annealing treatment of 200 °C for 30 min. (C) Titled view of the Al concavities enriched with Ag particles. (D) Magnified tilted view (45° Angle) of prepared Al concavities with Ag NPs. (E) UV-VIS spectra, (F) particle size distribution analysis. The Inset image shows the part of the template where particle size distribution was carried out with the help of Image J.

Following the initial anodization step, the NAA is eliminated by immersing it in a mixture of chromic acid (1.8 %) and phosphoric acid (6 wt%) for 3 h at 70 °C. Following the ethanol rinse and sample drying, the second anodization stage is conducted for 3 h under identical temperature and applied voltage (195 V) conditions. Upon completion, a layer of self-ordered pores with a hexagonal pattern and consistent size is formed, as depicted in Figure 1A. To achieve the desired nanoconcave structure on the surface of the Al sheet, the NAA samples were submerged in a vigorously stirred solution consisting of chromic acid (1.8 %) and phosphoric acid (6 wt%) for 2 h at a temperature of 70 °C. A hexagonal distribution of nanoconcavities with dimple arrays and upright bumps at the connection zones between three adjoining nanoconcavities is achieved.

### 2.2 Ag NPs formation on Al nanoconcavities substrates

This description outlines a straightforward and fast method for creating Au nanopatterns on hexagonally distributed nano-concave Al arrays. This method does not require reagents and results in the production of a highly sensitive SERS substrate. Various lithography techniques have traditionally been used to create SERS substrates, but these approaches are hindered by their time-consuming processes, costly equipment, and complex operational requirements. We suggest a method that involves the process of sputtering followed by thermal annealing [46].

Firstly, a thin layer of Ag is applied onto the Al nanoconcavities template using a sputtering mechanism, without the need for any reagents, through a process called physical evaporation deposition (PVD). The PVD process involves the vaporization of the required material, the vapor’s transformation, and the vapor’s condensation onto the substrate’s surface in a vacuum environment. Magnetron sputtering is a physical vapor deposition (PVD) process that utilizes argon ions in the plasma to deposit target atoms onto the substrate surface via the collision of high-energy ions [47]. The main method used to deposit the thin Ag coating was RF magnetron sputtering, using a BESTEC magnetron sputtering system with a silver target 99.99 % pure. Certain parameters were set: a pressure of 3 mTorr, an argon flow rate of 20 sccm, and an RF power of 30 W. Several sputtering durations and thermal annealing parameters were used to investigate the impact of these parameters on the resulting substrates. Varying arrangements of silver NPs were achieved on Al nanoconcave templates, depending on the duration of the sputtering and the time and temperature of annealing.

## 3 Results and discussion

We followed the preparation of the aluminum substrates by subjecting them to various instances associated with Ag sputtering and thermal annealing. The images of the different resulting templates may be seen in the SI (Figs. S1 to S5), where the different configurations of silver nanofractals in nanoconcave morphologies are demonstrated to depend on the sputtering time and the thermal annealing temperature. The prepared templates presenting a no homogeneous distribution of Ag NP were discarded for SERS measurements. It can be challenging to precisely measure analytes, get inconsistent signal amplification, and deal with poor reproducibility when Ag NPs are not distributed uniformly on an SERS substrate [48], [49].

In our study, to ensure consistent SERS activity, the optimum SERS substrate with a homogeneous distribution of Ag patterns across the substrate was obtained with a sputtering time of 180 s and a thermal annealing period of 200 °C for 30 min (Al-Con_Ag_180s_). This is because the size and interparticle distances are much more efficient at these parameters, contributing to SERS enhancement. Further conditions of sputtering and thermal annealing are discussed in the SI. The EDS quantitative analysis of this substrate is exhibited in Fig. S6. The FESEM images of the different formation steps of this defect-free morphology template can be observed in Figure 1.

The synthesis of NPs in the Al nanoconcavities is driven by the principle of surface energy minimization, as discussed previously for similar morphologies [50]. The morphology of the template and the thickness of the Ag layer deposited are crucial criteria in the synthesis of Ag NPs because these factors determine the size, distribution, and homogeneity of the produced NPs. Coalescence and migration of Ag NPs or atoms (Ostwald ripening) are both processes that occur during thermal annealing treatment [51]. The merging of two nanostructures into a single, massive particle is known as coalescence [52]. If the drop in primary system energy is sufficient, NPs may coalesce and travel to the substrate’s surface during particle conjugation. However, Ostwald ripening occurs when atoms are transferred from one nanostructure to another via surface diffusion or evaporation [53]. Mass transfer occurs independently of particle contact, in this case, thanks to decreased surface energy. So, both nanostructures can trade atoms with one another, forming smaller NPs through atom depletion and larger ones through atom accumulation. Thermal annealing’s foundational technique for surface-supported, well-separated metal NPs is the Ostwald ripening process, whereas coalescence may denote dense clusters. The topography of the nanoconcavities reacts with the Ag layer when the Al nanoconcavities that have been sputtered with Ag undergo thermal annealing. According to surface energy minimization theory, the creation of particles in hemisphere concavities is driven by a specific force [54]. The local excess chemical potential can be reduced by spreading the Ag atoms away from the protrusion to the concavity, which minimises the local curvature and surface energy, according to the Gibbs-Thomson equation Δµ = кγΩ, where ‘Ω’ is the atomic volume, γ is the surface energy, and ‘к’ is the local curvature [55]. It has been noted that the development of deposited nanostructures is greatly influenced by the sputtering period and annealing conditions. The geometry and quantity of particles change as the sputtering duration increases. Various NPs can be created and even merged near bigger particles, as demonstrated in Figure 1B; when thermal annealing at 200 °C is applied for 30 min, intriguing Ag structures are formed inside the nanoconcavities. Additionally, the magnified and tilted view of prepared Al concavities is shown in Figure 1C and D to enhance the understanding of particle distribution across the surface. This nano-structured platform is ideal for plasmon resonance applications because each cell has multiple Ag NPs [56]. The film morphology, deposition time, and thickness must create comparable nanostructures with the specified template configuration. The film morphology changes depending on the method and the conditions at hand. As demonstrated in this preliminary step, the Ag-enriched nanostructure was formed, which may guide the subsequent stages in measuring the characterization techniques and SERS activities. The Ag layer interacts with the surface features of the generated nanoconcavities when the honeycomb-like nanoarrays plated with Ag are exposed to thermal annealing. The deposition process and thermal annealing treatment were iterated with various parameters to achieve the target increase in particle size and decrease in interparticle distance. Reducing the distance between particles is crucial to attain interparticle plasmon interaction. An enhanced SERS signal or a more sensitive LSPR to refractive index variations may result from the strengthened coupling of the near field [57]. Bunches of particles of different sizes appear present in the distribution of Ag-formed particles and the aggregation of smaller particles. Silver NPs (AgNPs) can have their growth rate and properties drastically changed by heat annealing, particle size and morphology, growth rate, surface smoothing, improved crystallinity, increased agglomeration, shift in plasmon resonance, enhanced absorption and scattering, and thermal stability [58]. Because of these changes, the growth rate of Ag has practical consequences that can affect many applications; for example, imaging, sensors, and surface-enhanced Raman spectroscopy are all made possible by Ag’s distinctive plasmonic activity in smaller grains [59], [60]. Several forces and interactions contributed to the adherence of the Ag NPs to the Al concavities. One of these forces, the Van der Waals force, played a crucial role in the initial attachment of the NPs [61]. Through stamping or deposition, physical pressure determines the close interaction between the Al surface and NPs, and physical contact is also involved. This interaction is caused by several variables that vary with the preparation, including ligands and stabilizing agents, hydrophilic and hydrophobic contacts, electrostatic interactions, chemisorption, and surface functional groups [62], [63].

Ultraspectroscopy is an essential instrument for understanding the optical characteristics of Ag NPs bound to the concave surface of Al. Consequently, the UV spectra of the Al nanoconcave substrates with Ag sputtering, as shown in Figure 1E, exhibit wide peaks in the 400–450 nm band. A dynamic and changeable divergence exists between the vibrational and rotational levels. What makes UV-VIS spectroscopy possible are electronic transitions associated with changes in vibration and rotation. Consequently, absorption occurs across a spectrum rather than at a single wavelength. This causes the Absorbance versus Wavelength figure to seem like a standard Gaussian distribution down to the broad peak. The unique optical signature of silver NPs can be better understood and utilized by plotting their UV–Vis curves. Ag NPs with peaks in the specified band increase electromagnetic fields in their close vicinity to facilitate SERS measurements. The particle size distribution analysis of the prepared Ag nanopattern on Al concavities was measured and exhibited in Figure 1F.

The formation of “super-lenses” – nano-concentrators and resonant amplifiers – is achieved by attaching silver NPs to the surface and along the Al nanoconcavities. The collective oscillation of free conduction electrons produced by the incident light’s fluctuating electromagnetic field is referred to as surface plasmons in the Mie theory [64].

### 3.1 SERS detection of Amx (using Al_Con_Ag_180s_)

**Figure 2:**
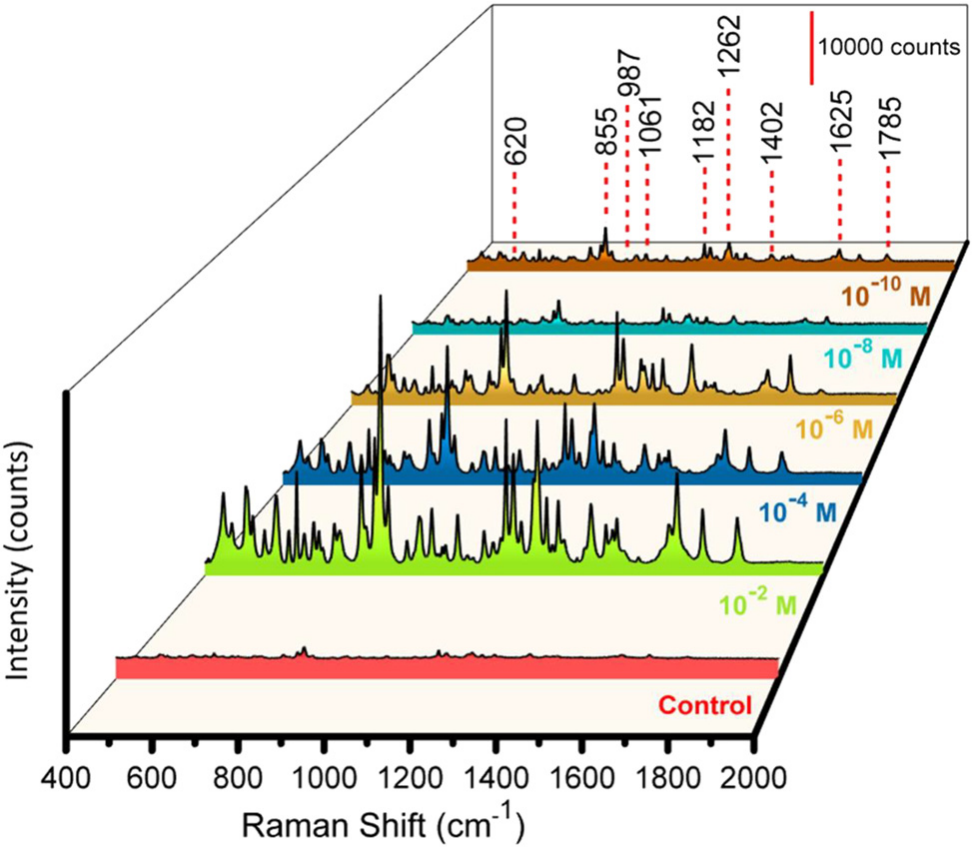
SERS measurements with substrate Al_Con_Ag_180s_ for detecting Amx at various concentrations (Control, and from 10^−2^ to 10^−10^ M), with multiple colors representing the different concentrations.

Amx has a molecular structure comprising a β-lactam ring, an amino group, and a phenyl group; it is an antibiotic that operates across various conditions. Its identification in pharmaceuticals and biological samples is vital for quality control and clinical diagnostics because of its widespread use to treat bacterial infections. An aqueous Amx solution was selected to measure the SERS activity of the prepared AgNPs Al nanoconcavities templates. The SERS detection of Amx with the prepared Al_Con_Ag_180s_ template is presented in Figure 2 for Amx concentrations ranging from 10^−2^ to 10^−10^ M. This figure shows that all the distinctive peaks of the Amx (Table 1) are identified, even for the lower concentration of Amx (10^−10^ M), increasing its intensity with increasing antibiotic concentrations. The control is an Amx concentration of 0.1 M on the nanoconcave Al template, but without Ag NPs. The enhancement in the Raman intensity of Amx using Ag NPs is significantly more apparent than that achieved with the antibiotic’s conventional Raman spectrum. The bands observed at 620 and 855 cm^−1^ are related to the δ(O13H) + δ (CH) + δ (CH_3_) and Ring2 breathing + δ(CH ring2) + δ(CH) + δ(N19H_2_). Amx’s δ(CH) + *v*(C16NH_2_) + δ(C15C16NH_2_) is observed at 987 cm^−1^. Additionally, at 1061 cm^−1^, *v*(C16NH_2_) + δ(NH_2_) + δ(CH) is observed. The vibrations associated with δ(CH ring2) + δ(O25H) + δ(CH) + δ(N19H_2_) are represented by the peak seen at 1182 cm^−1^. The *v*(C24OH) + δ(CH ring2) is represented by the peak that emerges at 1262 cm^−1^. The assignments τ(N19H_2_) + δ(C16H) and *v*(CC ring2) + δ(N19H) are denoted by the 1402 and 1625 cm^−1^. Additionally, the 1785 cm^−1^ peak represents the band assignment *v*(C8O14) + δ (C3H) + δ (O13H). The aromatic ring’s complete symmetric vibration, or its breathing vibration, typically appears as a strong band in Raman spectra. The estimated Raman spectrum for Amx shows this vibration at 855 cm^−1^. At 1625, 1262, and 1182 cm^−1^, respectively, the benzene ring and other strong Raman bands related to the benzene ring and C–H bending vibrations are visible in the Amx spectrum. These results are in accordance with previous studies reported in the literature about the Amx characteristic peaks [65]. The polarizability component changes perpendicular to the surface, which are the normal modes, are improved according to the SERS surface-selection principles. Theoretically, the interaction between Amx and the silver surface may be proven by integrating the p-electrons of rings’ addition to the lone pairs of electrons from the O, N, and S atoms [66]. To check the prepared substrate’s repeatability, Amx SERS measurements were carried out on various 4 points to get SERS signals, as shown in Fig. S7 (a). The sensor revealed good homogeneity for the detection of Amx. The SERS signals from several batches of our SERS substrates were analysed for a standard concentration of 10^−6^ M, to evaluate the batch-to-batch consistency, as shown in Fig. S7 (c). The sensing platform exhibits good consistency in Amx determination, as evidenced by the computed response RSD value from 0.7 to 2.8 %, depending on the characteristic peak.

**Table 1:** Amx’s experimental and theoretical vibrational frequencies and band allocations are reported in the literature and our work.

Literature Amx peaks (cm^−1^)	Our work (cm^−1^)	Band assignments
613, 616, 625	620	δ(O13H) + δ (CH) + δ (CH_3_)
852, 864	855	Ring2 breathing + δ (CH ring2) + δ (CH) + δ (N19H_2_)
972, 985	987	ρ (CH3) + op. Bending CH ring2
1044, 1052, 1069	1061	*v*(C16NH2) + δ (NH2) + δ (CH)
1163, 1177, 1159	1182	δ (CH ring2) + δ (O25H) + δ (CH) + δ (N19H_2_)
1239, 1257, 1259	1262	*v*(C24OH) + δ (CH ring2)
1355, 1374, 1396	1402	τ(N19H2) + δ (C16H)
1610, 1614, 1618	1625	*v*(CC ring2) + δ (N19H)
1774, 1793	1785	*v*(C8O14) + δ (C3H) + δ (O13H)

### 3.2 SERS detection of Tc using Al_Con_Ag_180s_

The Tc antibiotic family is famously effective against various bacteria, including those with a Gram-positive or -negative stain, spirochetes, obligatory intracellular bacteria, and protozoan parasites. Tc is a widely used antibiotic that can kill bacteria like chlamydia, acne, or travellers’ diarrhea. An aqueous solution of Tc was used to measure the SERS performance on prepared substrates. 0.1 M concentration of the Tc refers to the control. Further concentrations from 10^−2^ to 10^−8^ M are used to measure the SERS performance of the Al_Con_Ag_180s_ substrates. A concentration of Tc as low as 10^−8^ M is detected.

**Figure 3:**
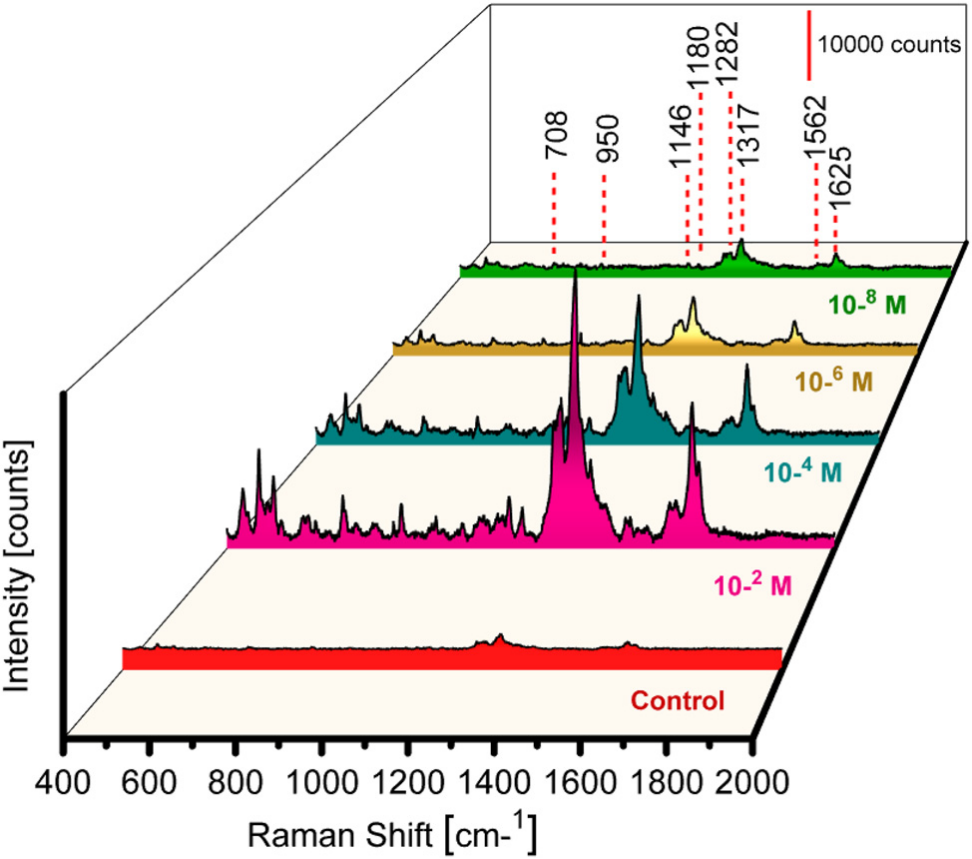
SERS measurements with substrate Al_Con_Ag_180s_ for detecting Tc at various concentrations (Control, and from 10^−2^ to 10^−8^ M), with multiple colors representing different concentrations.

Figure 3 displays various Tc Raman frequency bands, 1318−1655 cm^−1^ and 695−1298 cm^−1^. Several intense bands fill up the first region, such as the symmetric stretching of C21–C28 and C29–C31, the bending of C29–O32–H57 (1625 cm^−1^), and the rocking of C1–H33, C3–H37, and C8–H34 at 1317 cm^−1^. The second area shows Raman bands that are not as intense, such as the C8C11C19C24C21C15 ring breathing mode, C24–C29 and C19–O25 stretching at 1282 cm^−1^, C28–H54 rocking at 1180 cm^−1^, C2–C5 and C15–C22 stretching and C30–H55 rocking at 1148 cm^−1^, C21C24C29C31C30C28 ring deformation and C2 2H3 rocking at 948 cm^−1^, and C21C24C29C31C30C28 ring breathing and C22H3 rocking at 708 cm^−1^ [67]. Tc exhibits band shifting at several locations, such as 1625, 1180, 950, and 708 cm^−1^.

The amide group and secondary amine sites, the most hydrophilic and polar components of the molecule, are in the aromatic ring, so they are geographically isolated from hydrophobic sites [67], [68]. The repeatability measurement was studied by detecting Tc at 4 different points of the prepared substrate, as shown in Fig. S7 (b). To assess the batch-to-batch consistency, we produced several batches of our SERS substrates and measured the SERS signals for a standard concentration of 10^−6^ M, as illustrated in Fig. S7 (d). With a computed RSD value of 2.8–8.9 %, depending on the characteristic peak, the sensing platform sensor exhibits good consistency in determining Tc (Table 2).

**Table 2:** The literature and our work report Tc’s experimental and theoretical vibrational frequencies and band allocations.

Literature Tc peaks (cm^−1^)	Our work (cm^−1^)	Band assignments
695, 710	708	Breath ring C21C24C29C31C30C28, rock C22H3
936, 944	950	Def ring C21C24C29C31C30C28, rock C22H3
1122, 1139	1146	Str C2–C5, str C15–C22, rock C30–H55
1174, 1219	1180	Rock C28–H54
1288, 1293	1282	Breath ring C8C11C19C24C21C15, str C24– C29, str C19–O25
1316, 1319	1317	Rock C1–H33, rock C3–H37, rock C8–H34
1560	1562	C110 + (CC)D
1603, 1623	1625	Sym str C21–C28, C29–C31, bend C29–O32–H57

### 3.3 Multiplex SERS measurement of mixture (Amx + Tc)

**Figure 4:**
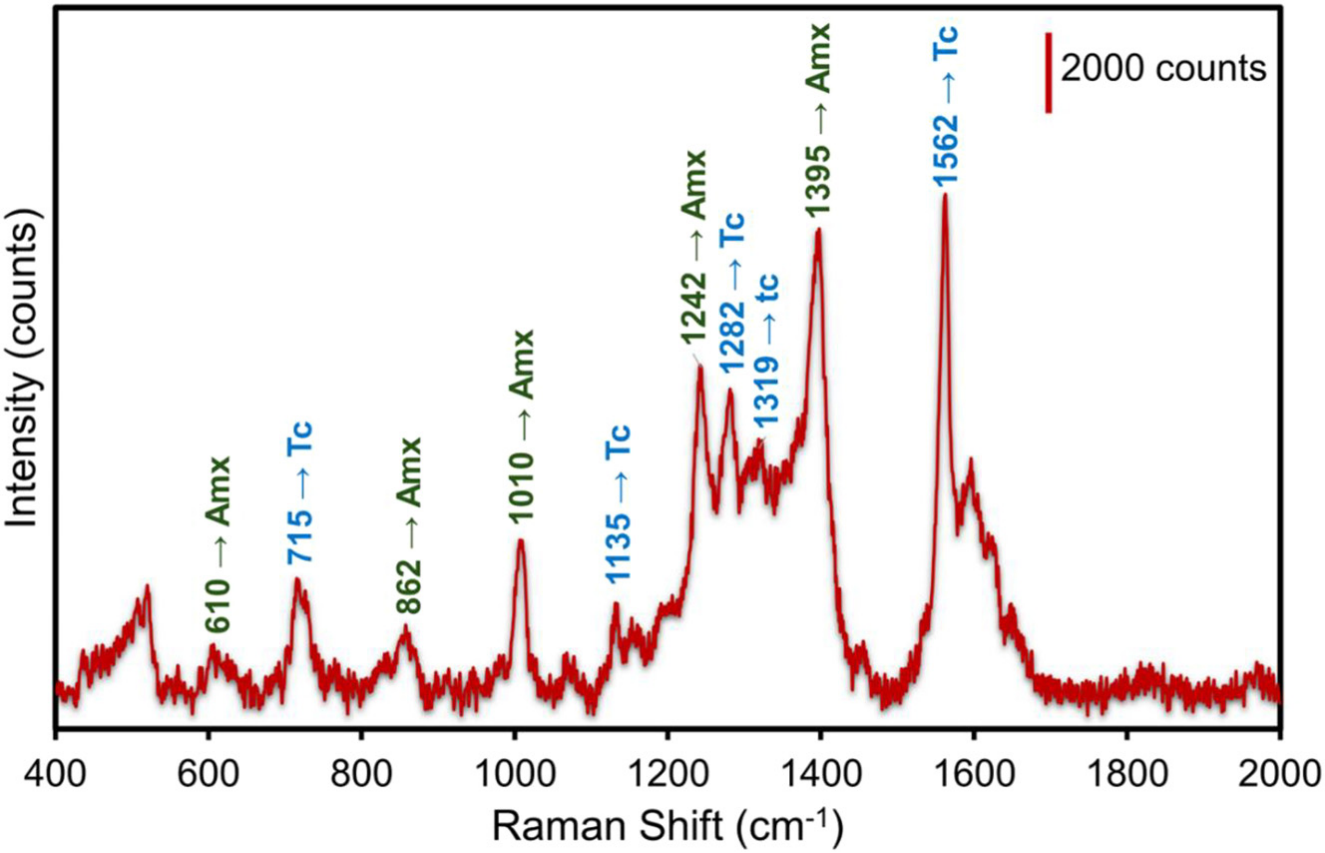
Multiplex SERS measurements of the mixture Amx + Tc for 10^−4^ M concentration using the Al_Con_Ag_180s_ template. The values in green color indicate the band assignments identifying Amx peaks. The values in blue indicate the band assignments identifying Tc peaks.

To assess the SERS efficacy in the simultaneous detection of antibiotics, we combined the solutions of Amx and Tc in a 1:1 ratio. The Amx and Tc solutions were thoroughly mixed at 10^−4^ M concentrations. The SERS spectrum obtained from the measurement of the Al_Con_Ag_180s_ with the Amx and Tc mixing is shown in Figure 4. Distinguishable peaks in the Raman spectra of Amx and Tc can be utilized for identification. Peaks identifying the presence of Amx are observed at 610, 862, 1010, 1242, 1395 cm^−1^ and those identifying the presence of Tc are observed at 715, 1135, 1282, 1319 and 1562 cm^−1^. These Raman peaks and their band allocations identifying each antibiotic of the mixture of the Amx and Tc are presented in Table 3. The combination of interactions of the medications with silver nanoparticles and the reciprocal effects of the antibiotics themselves are responsible for the peak shift observed in Table 3. Competitive adsorption, Antibiotic concentrations, intermolecular interactions, modifications in surface plasmon coupling, and conformational changes are the main causes of the peak shifts seen in SERS measurements of amoxicillin and tetracycline combinations [69]. For accurate SERS detection of many analytes it is essential to comprehend these mechanisms. Nevertheless, spectrum overlap may occur, which could result in interference. According to our findings, there was so very little interference between the SERS signals for the two medications, that did not affect the identification of the two antibiotics. The synergistic effect of silver nanoparticles and aluminum nanopits is the enhancing mechanism in our SERS substrates that contributes to the high sensitivity and selectivity [70]. This method preserves the precision of detection by ensuring that there is minimal overlap between the signals from the two medications.

**Table 3:** The Raman peaks and their band allocations identify each antibiotic of the mixture of Amx and Tc in the SERS spectrum.

Antibiotic	Raman shifts (cm^−1^)	Band assignments
Amx	610	δ(O13H) + δ (CH) + δ (CH_3_)
	862	Ring2 breathing + δ (CH ring2) + δ (CH) + δ (N19H_2_)
	1010	*v*(C16NH_2_) + δ (NH_2_) + δ (CH)
	1242	*v*(C24OH) + δ (CH ring2)
	1395	τ(N19H_2_) + δ (C16H)
Tc	715	Breath ring C21C24C29C31C30C28, rock C22H_3_
	1135	Str C2–C5, str C15–C22, rock C30–H55
	1282	Breath ring C8C11C19C24C21C15, str C24– C29, str C19–O25
	1319	Rock C1–H33, rock C3–H37, rock C8–H34
	1562	C110 + (CC)D

### 3.4 Sensing performance of the SERS platforms

Subsequent research examined how well the SERS signal magnification worked with the developed Ag-decorated substrates. Computing the enhancement factor (EF) as an objective measure of SERS proficiency is common practice. We used the standard approach, as shown in Equation (1) [71].

(1) $$\text{EF }=\;\left({\text{I}}_{\text{surf}}/{\text{N}}_{\text{surf}}\right)\left({\text{I}}_{\text{bulk}}/{\text{N}}_{\text{bulk}}\right)$$

whereas the intensities of the vibrational modes in the SERS and Raman spectra are denoted as I_surf_ and I_bulk_, respectively. N_bulk_ is the total number of molecules subjected to Raman spectrum analysis, and Nsurf is the number of molecules subjected to SERS.

The calculated EF of the Ag-decorated SERS platform for the detection of Amx is 8.9 × 10^8^ for the 855 cm^−1^ peak (concentration of 10^−10^ M). This peak shows a strong intensity and does not overlap with other peaks. For Tc detection, the calculated EF is 1.6 × 10^6^ (1318 cm^−1^ Raman shift). Both chemical (CM) and electromagnetic (EM) enhancement mechanisms are part of the complex SERS enhancement mechanism. The local electromagnetic field is strengthened by the rough surface created by the aluminum nanopits. These hot areas become even more intense when mixed with silver nanoparticles, which significantly improves the Raman signals. The enhancement of aluminum substrates may be facilitated by the transfer of charge between the metal’s Fermi level and the analyte molecules’ highest occupied molecular orbital (HOMO). Silver nanoparticles and aluminum nanopits work together to produce a synergistic effect that amplifies SERS signals. The silver nanoparticles function as effective nanoantenna’s, converting incident light into localized surface plasmon resonances (LSPRs), while the aluminum nano-pits offer a stable and textured basis [58], [72]. To achieve excellent sensitivity and reproducibility, our SERS substrates combine silver nanoparticles with aluminum nanopits, utilizing both chemical and electromagnetic enhancing methods. This synergistic impact is necessary for the substantial Raman signal amplification that we obtained in our study.

**Figure 5:**
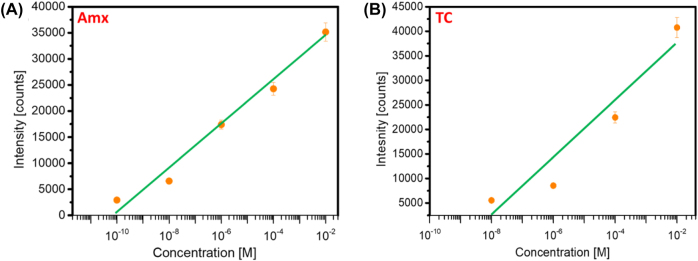
Direct correlation between the Raman intensity and the concentration of antibiotics (a) Amx and (b) Tc. The data points correspond to the measured Raman intensities at 855 cm^−1^ for Amx and 1317 cm^−1^ for Tc at different concentrations.

The sensing response of the developed SERS platform is studied in Figure 5. A linear response of the Raman intensity is observed for increasing concentrations of Amx (Figure 5A) and Tc (Figure 5B). This linearity is observed for both antibiotics in the range of concentrations evaluated for each. The limit of detection (LOD) of prepared AgNPs has been calculated using the expression [73], [74], [75]:

(2) $$\text{LOD }=\;3\text{.}3\left(Sy/S\right)$$

where “S*y*” is the Standard deviation, and S is the slope of the calibration curve. From Figure 5, the LOD for Amx is 2.5 × 10^−11^ M, and the LOD for Tc is 1.3 × 10^−9^ M. These results are coherent with the experimental values, as lower concentrations of Amx (10^−10^ M) have been detected (Figure 3) than for Tc (10^−8^ M). Both are within the range of the theoretical LOD values. These LODs obtained for Amx and Tc fall within the ranges of other methods reported in the literature for detecting the same antibiotics (see Table 4). The SERS enhancement factor is mostly determined by the size distribution of the nanoparticles. Research has demonstrated that more consistent and repeatable SERS signals might result from a smaller size dispersion. Another important element that has an impact on SERS performance is surface roughness. Higher SERS enhancement can result from rough surfaces because they can produce more “hot spots” where the electromagnetic field is concentrated [63], [70]. We offer a more thorough theoretical explanation for the high EF and low detection limit seen in our SERS substrates by examining the size distribution of the nanoparticles and surface roughness. Our substrates are extremely sensitive and repeatable for the detection of antibiotics due to the synergistic interaction between the silver nanoparticles and aluminum nanopits, which further improves the SERS performance.

**Table 4:** Comparison of the LOD of other methods reported to detect Amx and Tc.

SERS substrate	SERS substrate	Literature
Limit of detection (Amx)	Limit of detection (Tc)	
Ag-decorated polymeric microbeads	Substrate of Ag nanoparticle	[67], [76]
1.0 × 10^−8^ M	1 × 10^−9^ M	
Ag colloids	Fluorescent DPA-Ce-GMP-Eu	[77], [78]
50 × 10^−3^ M	6.6 × 10^−9^ M	
Ag nanocubes	Blue fluorescent carbon dots	[79], [80]
0.041 × 10^−9^ M	9.5 × 10^−8^ M	
Querecetagetin coated AgNPs	Carbon dots-doped lanthanide	[81], [82]
4.46 × 10^−6^ M	3.64 × 10^−9^ M	
ZnO NRs/gold/glass electrode	Natural nano-clay and carbon dots	[83], [84]
1.5 × 10^−5^ M	8.7 × 10^−9^ M	
AuNPs/en-MWCNTs	AuNP-coated MIOPPy matrix	[85], [86]
1.5 × 10^−8^ M	6.5 × 10^−7^ M	
**AgNPs on Al nanoconcavities**	**AgNPs on Al nanoconcavities**	**This work**
**2.5 ×** **10** ^**−**^ **^11^ M (Amx)**	**1.3 ×** **10** ^**−**^ **^9^** **M (Tc)**	

## 4 Conclusions

Our approach of altering ordered vertically arrayed nanostructures with varying silver NPs morphologies and adjustable plasmonic characteristics is simple and easy to implement. The SERS activity in detecting two common antibiotics, Amx, and Tc, was examined using the straightforward approach to producing the aluminum morphology enriched with silver NPs. The uniform surface structure of the aluminum substrate was accomplished utilizing a two-step anodization process, followed by chemical etching of alumina and thermal annealing of the dissolving silver layer. Results showed that SERS activity was well-detectable on the produced substrates, even at modest concentrations and with a combination of the two antibiotics. With its stable, reproducible, and highly functionalized array-based morphology, surface-enhanced Raman Spectroscopy SERS can be used for environmental monitoring, food safety investigations, and rapid identification of various probe molecules. The optical engineerable and reproducible aluminum-based templates’ individual and mixture antibiotic detection capabilities demonstrated encouraging SERS performance. These results pave the way for the simultaneous identification of several chemicals in any given solution.

## 5 Supplementary information

Find out more about the effects of varying sputtering temperatures and durations on prepared templates, how to determine the enhancement factor, and investigate the morphology of the Ag patterns produced on Al nanoconcavities in the supplementary materials.

## Supplementary Material

Supplementary Material Details
