# Supplementary material for: Self-ordered silver nanoparticles on nanoconcave plasmonic lattices for SERS multi-antibiotic detection
Source: Nanophotonics. 2025 Jun 26;14(15):2605–18. doi: 10.1515/nanoph-2025-0108 (PMC12322726; doi:10.1515/nanoph-2025-0108)
Supplement: Supplementary file 1 — Supplementary Material Details [file j_nanoph-2025-0108_suppl_001.pdf]

Supporting Information

Self-ordered Silver Nanoparticles on Nanoconcave Plasmonic Lattices for SERS Multi-Antibiotic Detection

Gohar Ijaz Dar, Elisabet Xifre-Perez*, and Lluis F. Marsal*

Departament d'Enginyeria Electrònica, Elèctrica i Automàtica, Universitat Rovira i Virgili,

Avinguda Països Catalans 26, 43007 Tarragona, Spain,

Email: eliosabet.xifre@urv.cat, lluis.marsal@urv.cat

MATERIALS

Aluminum (Al) foils with a thickness of 0.32 millimetres and a high purity of 99.9997 percent were acquired from Goodfellow from Cambridge Ltd. in the United Kingdom. The following substances were supplied by Sigma Aldrich: phosphoric acid (H_3PO_4; purity 97%), perchloric acid ($HClO_4$), chromic acid (H_2CrO_4), hydrochloric acid (HCl), Amoxicillin, ethanol (C_2H_5OH, EtOH) and tetracycline. The preparation of aqueous solutions was accomplished by using ultrapure water.

CHARACTERIZATION TECHNIQUES

Thermo Fisher Scientific's Scios 2 field-emission scanning electron microscope (FESEM) with a 5 kV acceleration voltage. Using a 785 nm excitation laser, 50X LWD objective lens, and 1200 lines/mm grating, SERS data were acquired using the confocal Raman micro spectrometer (Renishaw inVia Raman Microscope). A 10-second exposure was used to measure each substrate, and the laser power was 10 mW. The laser point measured 1 μm in diameter.

AG NANOFRACTALS FORMATION ON ALUMINUM CONCAVITIES

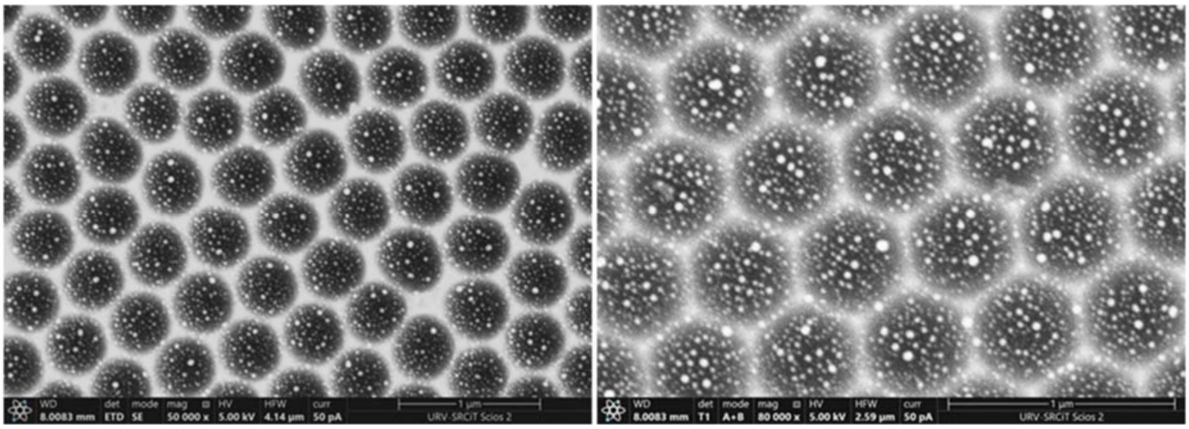

Figure S1. Aluminum nanoconcavities decorated with 100 s of silver sputtering followed by thermal annealing of 200° C for 30 min.

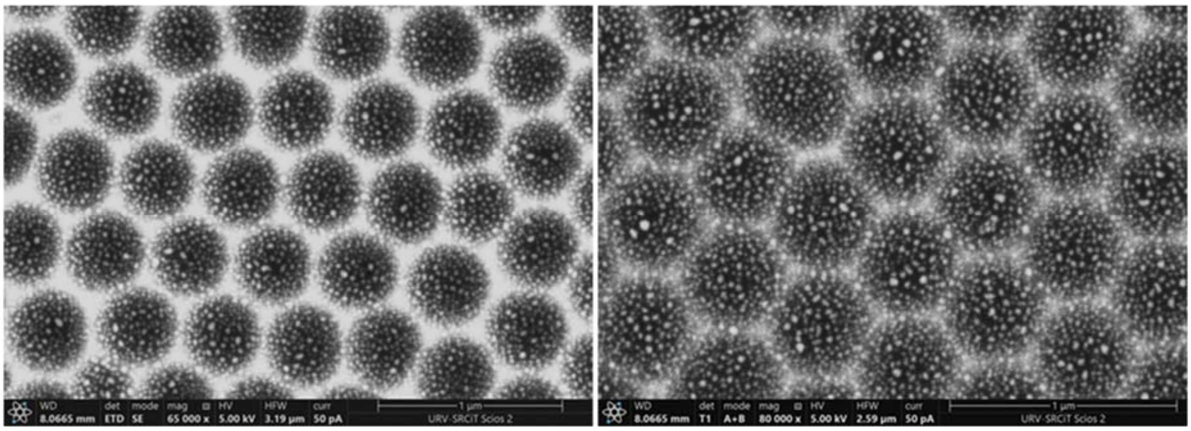

Figure S2. Aluminum naoconcavities decorated with 150 s of silver sputtering followed by thermal annealing of 200° C for 30 min.

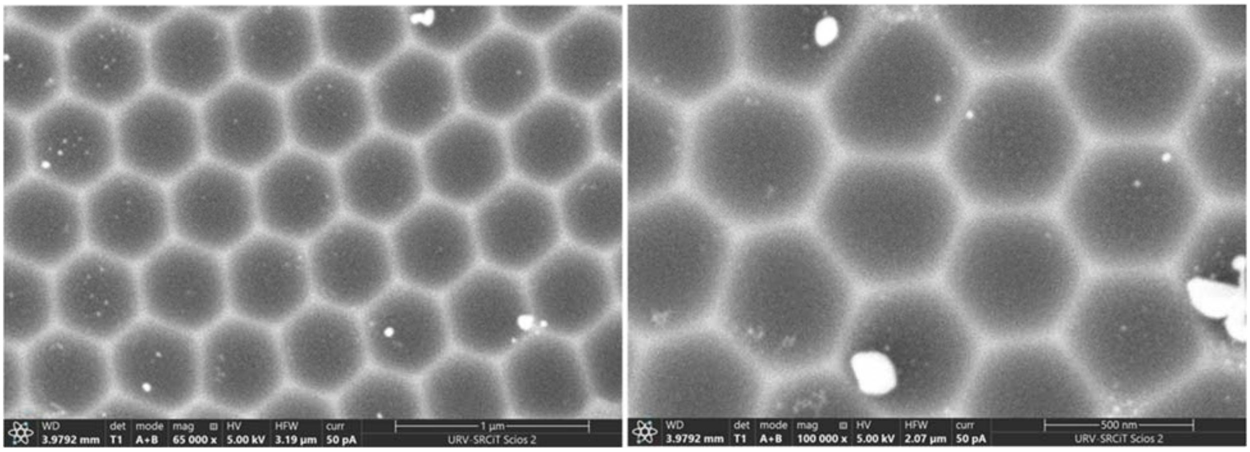

Figure S3. Aluminum nanoconcavities decorated with 100 s of silver sputtering followed by thermal annealing of 300° C for 30 min.

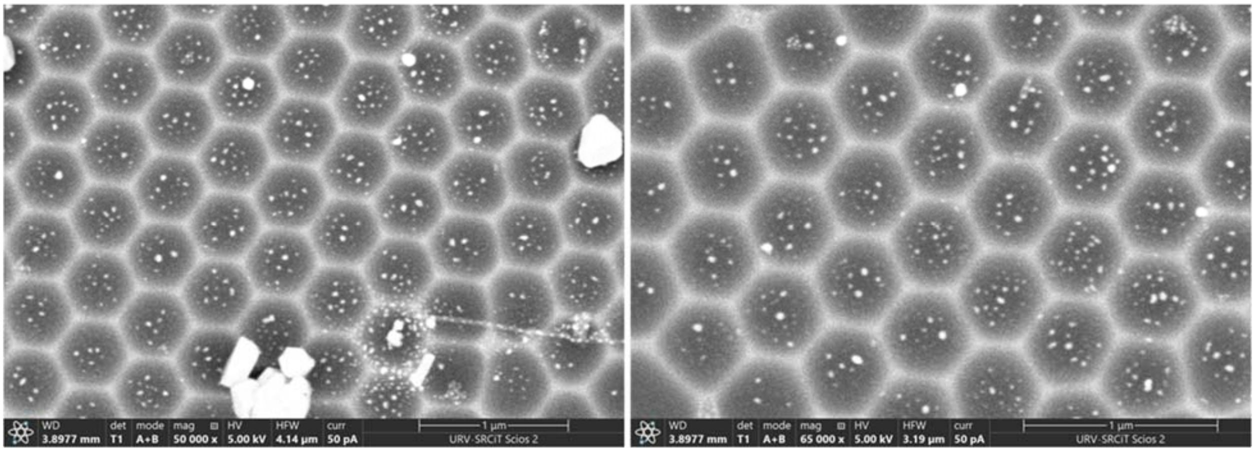

Figure S4. Aluminum nanoconcavities decorated with 150 s of silver sputtering followed by thermal annealing of 300° C for 30 min.

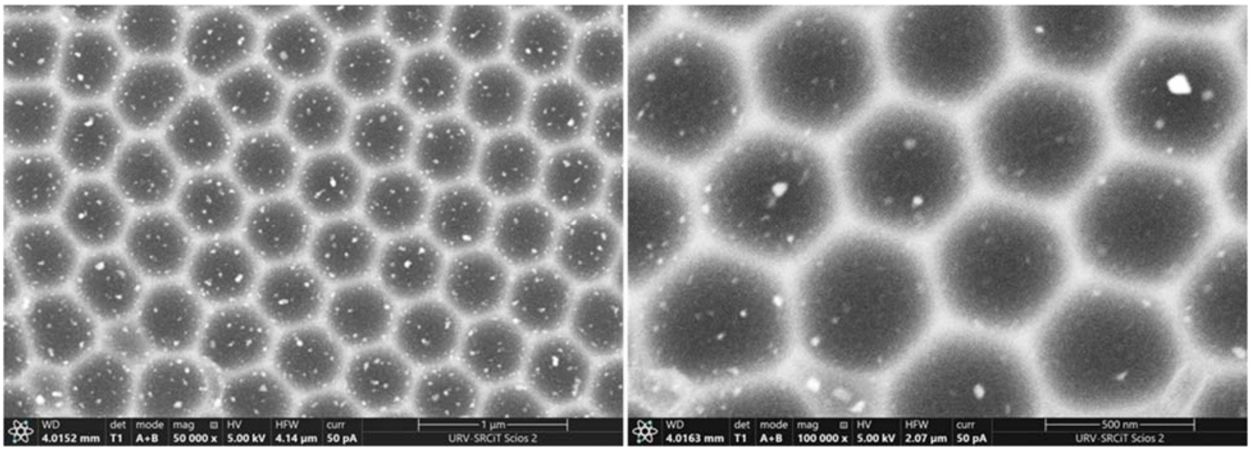

Figure S5. Aluminum nanoconcavities decorated with 200 s of silver sputtering followed by thermal annealing of 300° C for 30 min.

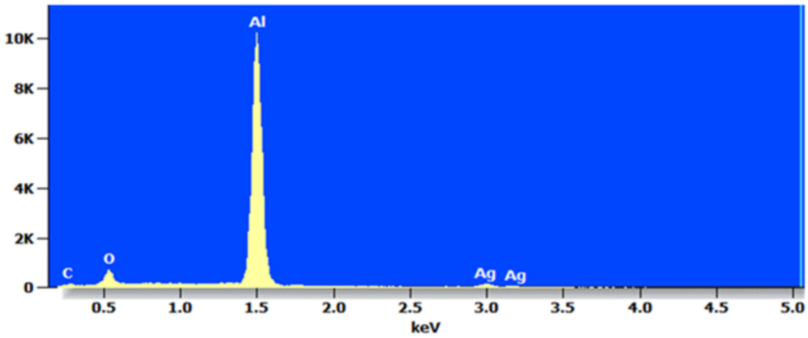

Elements	Weight %	Weight % err	Atom %	Norm. Wt. %	Chemical Formula
C K	0.64	0.03	1.68	0.64	C
O K	3.35	0.06	6.56	3.35	O
Al K	73.24	0.35	85.14	73.24	Al
Ag L	24.77	1.00	6.62	22.77	Ag
	100.00		100.00	100.00	

Figure S6. Quantitative EDS examination of the resulted Ag enriched nanofractals for the The Al substrate with 180 s sputtering and thermal sintering at 200° C for 30 minutes (Al-Con_$Ag_{180\ s}$)

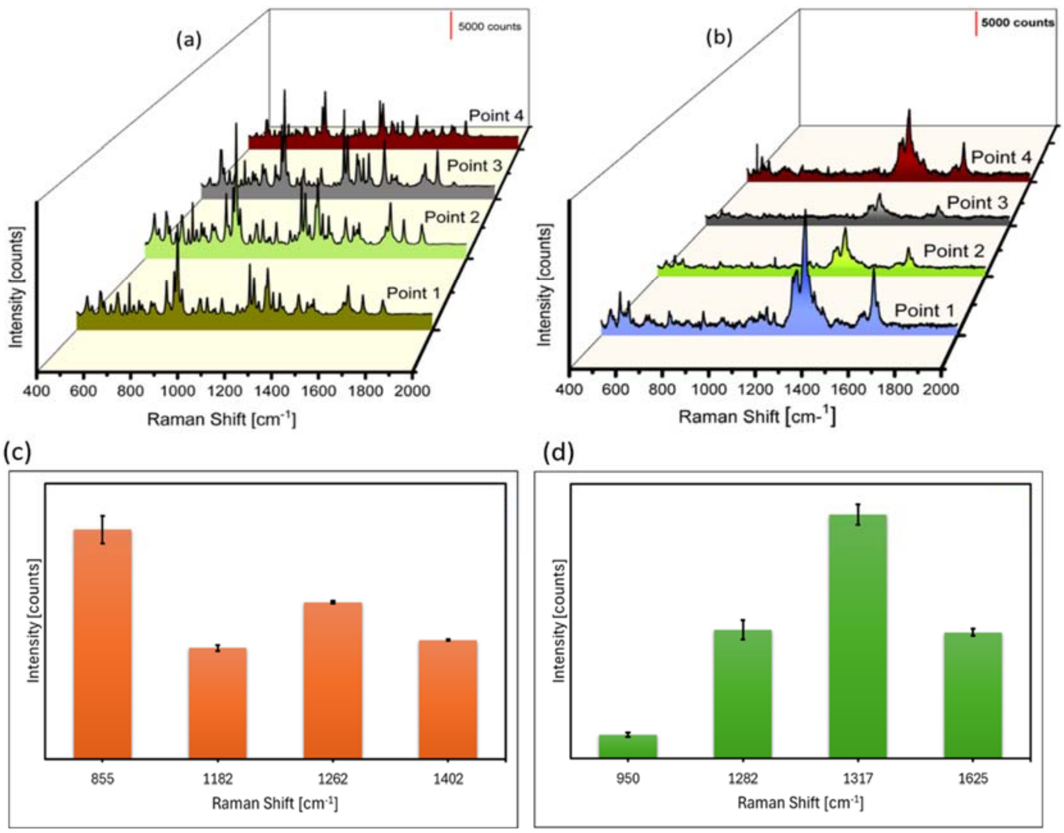

Figure S7. Raman spectra of different points of the same substrate for the detection of (a) amoxicillin (10^{-6} M) and (b) tetracycline (10^{-6} M). SERS intensity at different characteristic Raman peaks for different batches for the detection of the amoxicillin (c) and tetracycline (d).
